# MoS_2_-Decorated Graphene@porous Carbon Nanofiber Anodes via Centrifugal Spinning

**DOI:** 10.3390/nano12142505

**Published:** 2022-07-21

**Authors:** Elham Abdolrazzaghian, Jiadeng Zhu, Juran Kim, Meltem Yanilmaz

**Affiliations:** 1 Department of Nano Science and Nano Engineering, Istanbul Technical University, Istanbul 34469, Turkey; elham.a1992@gmail.com; 2 Oak Ridge National Laboratory, Chemical Sciences Division, Oak Ridge, TN 37831, USA; jzhu14@ncsu.edu; 3 Advanced Textile R&D Department, Korea Institute of Industrial Technology (KITECH), Ansan 15588, Korea; 4 Department of Textile Engineering, Istanbul Technical University, Istanbul 34437, Turkey

**Keywords:** carbon, nanofibers, anodes, polyacrylonitrile

## Abstract

Sodium-ion batteries (SIBs) are promising alternatives to lithium-ion batteries as green energy storage devices because of their similar working principles and the abundance of low-cost sodium resources. Nanostructured carbon materials are attracting great interest as high-performance anodes for SIBs. Herein, a simple and fast technique to prepare carbon nanofibers (CNFs) is presented, and the effects of carbonization conditions on the morphology and electrochemical properties of CNF anodes in Li- and Na-ion batteries are investigated. Porous CNFs containing graphene were fabricated via centrifugal spinning, and MoS_2_ were decorated on graphene-included porous CNFs via hydrothermal synthesis. The effect of MoS_2_ on the morphology and the electrode performance was examined in detail. The results showed that the combination of centrifugal spinning, hydrothermal synthesis, and heat treatment is an efficient way to fabricate high-performance electrodes for rechargeable batteries. Furthermore, CNFs fabricated at a carbonization temperature of 800 °C delivered the highest capacity, and the addition of MoS_2_ improved the reversible capacity up to 860 mAh/g and 455 mAh/g for Li- and Na-ion batteries, respectively. A specific capacity of over 380 mAh/g was observed even at a high current density of 1 A/g. Centrifugal spinning and hydrothermal synthesis allowed for the fabrication of high-performance electrodes for sodium ion batteries.

## 1. Introduction

Green energy storage technology is receiving significant attention owing to the increasing effects of global warming. To decrease the negative effects of greenhouse gases on the environment, the usage and storage of green energy must increase [1,2,3]. Because of their high energy density, low self-discharge rate, light weight, fast charging speed, and long battery life, lithium-ion batteries (LIBs) are one of the most prominent energy storage devices, with many applications in daily life including portable electronics and electric vehicles. Owing to limited lithium sources and its high cost, sodium-ion batteries (SIBs) have been presented as an alternative energy storage technology. SIBs have received considerable attention because of their low cost and abundant sodium sources [4,5]. However, SIBs suffer from increased volume expansion, low energy density, and inferior cycling stability, due to the larger ionic radius of ${\mathrm{Na}}^{+}$ (${\mathrm{Na}}^{+}$: 1.02 Å vs. ${\mathrm{Li}}^{+}$: 0.76 Å). Considering these drawbacks, finding an appropriate electrode material with high specific capacity and long cycling life is critical [1,6,7].

Carbonaceous materials, such as graphene, hard carbon, carbon nanofibers (CNFs), carbon sheets, and carbon nanotubes, are abundant and inexpensive; thus, nanostructured carbons are the most favorable materials for use as SIB anodes [8]. Because CNFs are amorphous carbons with a one-dimensional structure, high surface area to volume ratio, high temperature resistance, and good electrical/thermal conductivity, they can provide good access to electrolytes and short transport distances for ${\mathrm{Na}}^{+}$ ions [1,4]. For CNFs, the short transport lengths for ions in the electrode and well-interconnected structure provide a continuous electron pathway, yielding fast transport channels; thus, ions in electrolytes have easy access to the porous, 3D-structured electrodes [9].

Porous nanostructured carbons are important materials, particularly for sensors, fuel cells, and energy storage, because of their high surface areas [6]. Additionally, porous CNFs are one of the most practical electrodes for SIBs because of their high surface area and large number of defects on the nanofiber surfaces, which facilitate better contact with the electrolyte and promote the transfer of ions/electrons. To synthesize porous CNFs, pore generators, such as polymethyl methacrylate (PMMA), polystyrene (PS), Pluronic F-127, and polytetrafluoroethylene (PTFE), could be used [1].

Graphene with a two-dimensional honeycomb structure is commonly used in energy storage devices because of its high theoretical specific capacity, large specific surface area, and good electronic conductivity, which can decrease the volume expansion during the charge/discharge process. Kim et al. synthesized a hybrid CNF/highly branched graphene nanosheet (HBGN) via a chemical vapor deposition method and used it as an electrode in LIBs. The HBGN delivered a reversible capacity of approximately 300 mAh/g after 200 cycles and showed high energy density, electrical conductivity, and chemical stability because of the structure of HBGN with its exposed edges that provided more Li storage sites, large surface areas, and more cavities [10]. Dufficy and co-workers [11] fabricated thermally reduced graphene oxide-containing CNFs (TRGO/CNFs) via an electrospinning method followed by heat treatment and then used it as an anode in LIBs. They compared TRGO/CNFs with pure CNF without GO and reported that CNFs containing 5 wt.% GO delivered a high discharge capacity of 830 mAh/g in the first cycle, while CNFs delivered a capacity of 595 mAh/g. This demonstrated that the addition of TRGO improved the specific capacity of CNFs by providing more space for Li-ion storage, shortening the pathway for Li-ion diffusion, and facilitating Li-ion transportation for charge transfer at fast charging rates [11].

The high capacity and graphene-like layered structure of MoS_2_ makes it a promising anode material for SIBs. However, capacity fading and poor capacity retention resulted from the large volume change and pulverization, which need to be adressed for practical applications. CNFs could buffer the volume change and improve the structural integrity of MoS_2_-based electrodes [12]. Yuan et al. [12] fabricated Mxene@MoS_2_@C electrodes with stable cycling performance owing to volume elasticity and excellent electrical conductivity of hierarchical Mxene and carbon structure. Li et al. [13] prepared graphene@MoS_2_ composite electrode and stable cycling performance was attributed to the conductive network, enhanced charge transfer, and alleviated volume expansion with the addition of graphene.

Although CNFs can be produced via different techniques, such as electrospinning, self-assembly, template synthesis, and chemical vapor deposition, centrifugal spinning has attracted significant attention owing to its high production rate, low cost, and reliable safety [14,15,16,17]. In this study, CNFs and MoS_2_-decorated graphene@porous CNFs were prepared for the first time by combining centrifugal spining, a novel, fast, and reliable production technique, hydrothermal synthesis, and heat treatment processing. The electrochemical properties could be optimized by tuning the thermal treatment conditions and thus controlling the structural parameters. The effects of carbonization temperature and MoS_2_ addition on the morphology and structure of the CNFs were studied using scanning electron microscopy (SEM), X-ray diffraction (XRD), and Raman spectroscopy. The electrochemical performance of the prepared CNFs and MoS_2_-decorated graphene@porous CNFs were also investigated as anodes in Li- and Na-ion batteries.

## 2. Materials and Methods

Polyacrylonitrile (PAN, Mw = 150,000), polystyrene (PS), N,N-dimethyl formamide (DMF), thiourea, ammonium molybdate tetrahydrate, and graphene were purchased from Sigma-Aldrich, St. Louis, MO, USA. PAN nanofibers were prepared by centrifugal spinning. A 10 wt.% solution was fed into the spinneret, and a rotational speed of 4000 rpm was applied. The spinneret-to-collector distance was set to 10 cm. To obtain CNFs and determine the optimum carbonization temperature, PAN nanofibers were stabilized at 280 °C for 3 h and carbonized at three different temperatures: 700 °C, 800 °C, and 900 °C. In addition, a PAN/PS/graphene solution was prepared to produce porous CNFs with enhanced specific surface area and improved conductivity. MoS_2_-decorated carbon nanofibers were synthesized via hydrothermal synthesis. A certain amount of thiourea and ammonium molybdate tetrahydrate was dissolved in distilled water, and PAN/PS/graphene nanofibers were immersed in this solution. The solution was then transferred to the hydrothermal reactor and heated up to 180 °C for 24 h. The as-prepared MoS_2_-decorated PAN/PS/graphene nanofibers were stabilized at 280 °C for 3 h and carbonized at 800 °C for 2 h. As-prepared CNFs were used as electrodes in Na-ion and Li-ion cells. Electrochemical experiments were performed using two electrode coin-type (316ss CR2032) half cells. In SIBs, a Whatman glass microfiber filter membrane was used as a separator, and sodium metal was used as the counter electrodes. The electrolyte of the SIBs was composed of a 1.0 M of NaClO_4_ solution in mixed ethylene carbonate (EC) and propylene carbonate (PC) at 1:1 by volume. Polypropylene membrane was used as a separator, and lithium metal was used as the counter electrodes in Li-ion batteries. The electrolyte of the LIBs was composed of a 1.0 M of LiPF_6_ solution in mixed ethylene carbonate (EC), dimethyl carbonate (DMC), and diethyl carbonate (DEC) at 1:1:1 by volume. A constant current was applied to the working electrode between two potential values. The electrochemical measurements were performed by carrying out galvanostatic charge/discharge tests of 0–2.5 V at 100 mA/g.

SEM (Zeiss Sigma 300, Oberkochen, Germany) was used to study the morphology of the nanofibers. The fiber diameters were calculated by measuring 100 randomly selected nanofibers in SEM images using Revolution software for each sample. XRD (PANalytical Empyrean, Malvern, UK) with a step of 0.01 and speed of 4° per minute and Raman spectroscopy (WITech alpha 300R, Ulm, Germany) were used to characterize the structure. The composition of MoS_2_-decorated graphene@porous CNFs were determined by thermogravimetric analysis (TGA, Hitachi, Tokyo, Japan). The sample was heated at a rate of 10 °C/min from room temperature to 600 °C in air. The cycling tests (Neware, Shenzhen, China) to determine the electrochemical performance were performed at room temperature.

## 3. Results

### 3.1. Morphology and Structural Characterization

**Figure 1:**
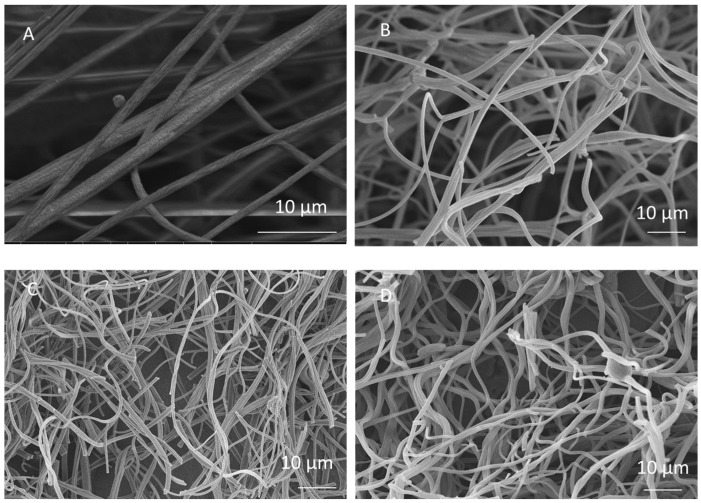
SEM images of (**A**) PAN nanofibers and carbon nanofibers carbonized at (**B**) 700 °C (**C**) 800 °C, and (**D**) 900 °C.

In this study, a centrifugal spinning system was used to fabricate PAN and graphene/PAN/PS nanofibers. A fibrous structure with an average fiber diameter of approximately 990 nm was observed in the SEM images of the PAN nanofibers, as shown in Figure 1A.

After spinning, PAN nanofibers are converted to CNFs by applying a two-step thermal treatment that includes stabilization and carbonization. In the stabilization process, the nanofibers are heated in air at 280 °C for 3 h. During this time, the chemical structure of PAN was changed to a ladder structure with cyclization, dehydrogenation, aromatization, oxidation, and crosslinking reactions occurring in the air atmosphere. Crosslinking occurs with the formation of triazine, which is the result of an intermolecular reaction involving three nitriles of three different PAN chains to prevent decomposition at high temperatures. Intermolecular and intramolecular nitrile crosslinking reactions are crucial for determining the final structure because these reactions change the chemical structure of PAN to form a ladder-like structure due to cyclization of the nitrile groups and thus determine the structure of the CNFs. An incomplete stabilization process leads to poor carbon properties. Following stabilization, PAN was carbonized in a N_2_ atmosphere at 700 °C, 800 °C, and 900 °C for 2 h to form polyaromatic (graphite structure) and disordered carbons (turbostratic structure). The carbonization process includes an aromatic growth and polymerization step, in which the noncarbon elements are removed as volatile gases in the form of hydrogen, nitrogen, water, ammonia, and so on [18,19].

SEM images of the CNFs carbonized at temperatures of 700 °C, 800 °C, and 900 °C are shown in Figure 1B–D. The average diameters of the CNFs were 930, 888, and 850 nm at 700 °C, 800 °C, and 900 °C, respectively. The average diameter of the nanofibers decreased after the carbonization process due to the removal of noncarbon elements during carbonization and decreased distances between the flat sheets of carbon [20]. In addition to reduced fiber diameters, a rough fiber surface was observed due to the presence of an amorphous structure and defects, which are beneficial for ion diffusion and increased contact area between the electrode and the electrolyte. With increasing carbonization temperature, most of the functional groups such as the nitrile group were eliminated, which led to a decrease in the average diameter of the fibers. Similar results were reported in previous studies. Lee et al. synthesized electrospun PAN-based activated CNFs and reported that the diameter of fibers decreased from 670 nm to 640 nm as the carbonization temperature increased from 800 °C to 950 °C [21]. Munajat et al. investigated the effects of carbonization temperature on the morphology of nanofibers produced by electrospinning and reported that the average diameter of CNFs decreased from 658.80 nm to 352.40 nm as the carbonization temperature increased from 800 °C to 1200 °C because of the removal of noncarbon elements from the structure of the nanofibers [22].

**Figure 2:**
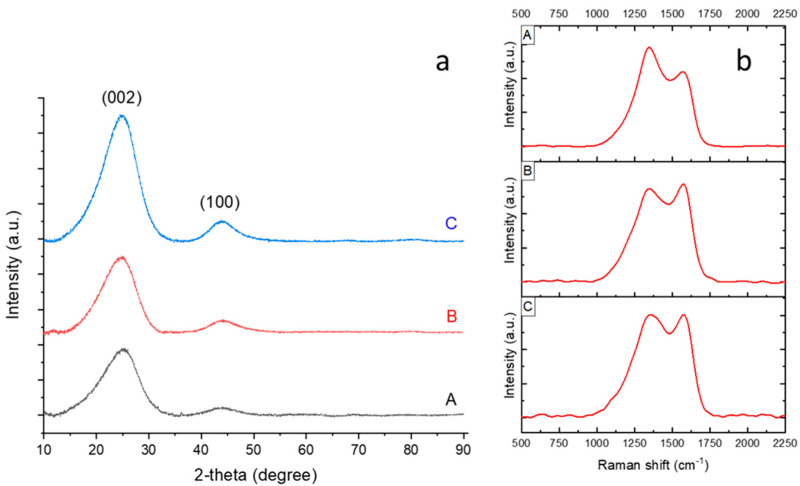
XRD pattern (**a**) and RAMAN spectra (**b**) of carbon nanofibers carbonized at (**A**) 700 °C (**B**) 800 °C, and (**C**) 900 °C.

The XRD pattern of the CNFs carbonized at different temperatures are shown in Figure 2a. All XRD patterns show a wide diffraction peak at approximately 25.0° corresponding to the (002) plane, which refers to the highly disordered structure of the CNFs [23]. The interlayer spacing, $d_{\left(002\right)}$, was determined using the Bragg equation λ = 2dsin θ, where λ is the wavelength of the incident X-ray source from Cu (λ = 1.5406 Å), and θ is the diffraction angle for the peak position. The d_(002)_ values of the CNFs carbonized at 700 °C, 800 °C, and 900 °C were calculated to be around 0.359, 0.353, 0.352 nm, respectively, which are larger than the d spacing of graphite (0.336 nm). As the carbonization temperature increased from 700 °C to 900 °C, the (002) peak sharpened, indicating structural development and a more ordered structure. Similar result was also reported by Choi et al. [24]. Qanati et al [19] and Zhou et al. [25] also reported decreased d value with increasing temperature and decreasing d spacing was explained by consolidated the sheets of carbon atoms, rearranged graphite crystallites and more graphitic structure with increased carbonization temperatures [19,25]. The carbonization temperature played an important role in this structural transformation. When the nanofibers were carbonized at higher temperatures, their chemical structure began to change, and some functional groups were removed, resulting in a more ordered structure [26]. When the carbonization temperature was 700 °C, the peak at 43° corresponding to the (100) graphite basal plane was slightly apparent, indicating the low crystalline structure of the carbon nanofibers [27]. As the temperature increased, the intensity of the peak at 43° increased owing to the more crystalline structure and partially graphitized carbon.

Raman spectra of the CNFs carbonized at 700 °C, 800 °C, and 900 °C are shown in Figure 2b(A–C), respectively. All studied CNFs demonstrated a strong peak at approximately 1350 ${\mathrm{cm}}^{-1}$, corresponding to the D band, which was attributed to the disordered carbon phase and structural defect sites. Another peak appearing at around 1570 ${\mathrm{cm}}^{-1}$ corresponds to the G band, which was attributed to the graphitic phase of the carbon [28]. As the carbonization temperature of the CNFs increased from 700 °C to 900 °C, the intensity of the G peak increased, and the structure of the CNFs became more ordered. This meant that the heat treatment decreased the disorders and defects of the CNFs, and the amount of graphitization increased with increasing temperature. Rajabpour et al. also reported a more ordered structure with increasing temperature [29]. The intensity ratio of the D band to the G band (R = I_D_/I_G_) reveals the quantity of ordered graphitic structure. As the temperature increased from 700 °C to 900 °C, R decreased from 1.10 to 0.99, indicating a more ordered structure at 900 °C. Zhang et al. [30] also reported that I_D_/I_G_ decreased as the carbonization temperature increased, which indicates that an increase in temperature improves the quality of the graphitic structure.

Both XRD and Raman data demonstrated that, as the carbonization temperature increased, the graphitization of the CNFs increased, and their structure became more ordered. In addition, the crystalline stacking size (L_c_) was determined using the Deby–Scherrer formula: kλ = L_c_·β·cosθ, where K is the Scherrer parameter (K = 0.94 for the (002) diffraction peak) [31], and β is the full-width at half-maximum (FWHM) in radians. As seen in Figure 2, the amount of L_c_ is 1.07, 1.11, and 1.13 for CNFs carbonized at 700 °C, 800 °C, and 900 °C, respectively. The in-plane graphitic crystallite size (L_a_) was calculated using the following equation: L_a_(nm) = 4.4(I_D_/I_G_)^−1^, which was developed by Knight and White [32]. There is an inverse relationship between L_a_ and I_D_/I_G_. With increasing temperature, the I_D_/I_G_ ratio decreased and L_a_ increased. The L_a_ values were 3.9, 4.43, and 4.43 nm as the temperature increased from 700 °C to 800 °C and then to 900 °C, respectively. Additionally, the area proportions (area under (002)/area under (100)) of CNF-700, CNF-800, and CNF-900 were 12.58, 9.06, and 8.55, respectively, demonstrating that, with increasing carbonization temperature, the structure of the CNFs became more ordered.

### 3.2. Electrochemical Evaluation of CNFs

**Figure 3:**
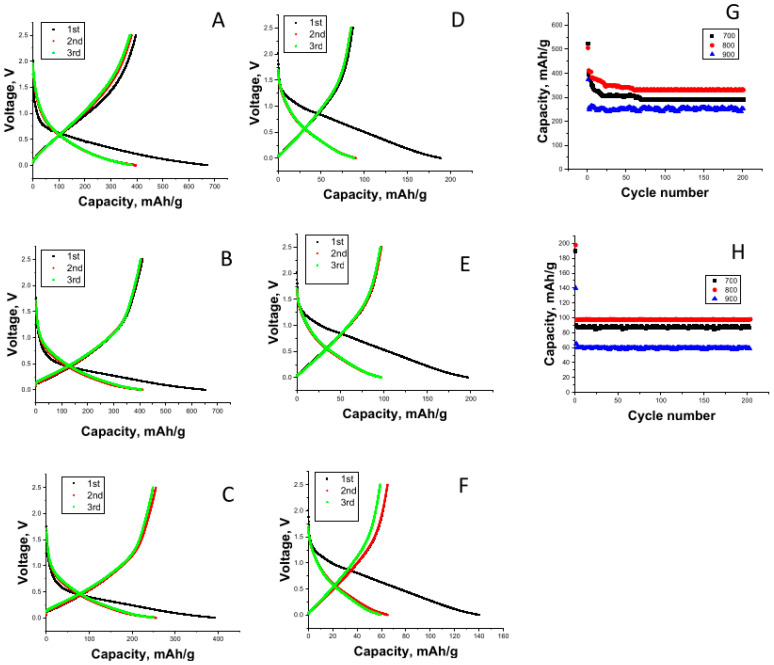
First-cycle charge/discharge curves of carbon nanofibers carbonized at (**A**,**D**) 700 °C, (**B**,**E**) 800 °C, and (**C**,**F**) 900 °C in Li-ion cells and Na-ion batteries and cycling performance in Li-ion batteries (**G**) and Na-ion batteries (**H**).

The first Li-ion cell discharge–charge curves for the CNFs carbonized at 700 °C, 800 °C, and 900 °C are shown in Figure 3A–C, respectively. The initial discharge capacities were 671, 652, and 392 mAh/g for the CNFs carbonized at 700 °C, 800 °C, and 900 °C, respectively. The charge capacities of the CNFs carbonized at 700 °C, 800 °C, and 900 °C were 396, 411, and 255 mAh/g, respectively. The initial irreversible capacity can be ascribed to the decomposition of the electrolyte components forming an solid electrolyte interphase (SEI) layer on the electrode surface [33].

CNFs were also used as electrodes in Na-ion cells, and the first discharge–charge curves for the CNFs carbonized at 700 °C, 800 °C, and 900 °C are shown in Figure 3D–F, respectively. The initial discharge capacities of the CNFs carbonized at 700 °C, 800 °C, and 900 °C were 189, 197, and 140 mAh/g, respectively. The charge capacities of the CNFs carbonized at 700 °C, 800 °C, and 900 °C were 86, 97, and 65 mAh/g, respectively. Similar to Li-ion cells, a large irreversible capacity was observed in the first cycle because of the decomposition of electrolyte components forming an SEI layer on the electrode surface.

The cycling performances of the Li-ion cells containing CNFs carbonized at 700 °C, 800 °C, and 900 °C are shown in Figure 3G. The reversible capacities of the CNFs carbonized at 700 °C, 800 °C, and 900 °C were respectively 290 mAh/g, 330 mAh/g, and 250 mAh/g in 200 cycles. Increasing carbonization temperature improves electrical conductivity, while a more disordered structure is beneficial for ion insertion [25]. Increasing the carbonization temperature from 700 °C to 800 °C resulted in a higher reversible capacity due to increased conductivity; however, further increasing the temperature to 900 °C resulted in a decrease in the reversible capacity because of the more ordered structure. Furthermore, the improved cycling performance of the CNFs carbonized at 800 °C was attributed to the highly disordered structure confirmed by Raman and XRD studies. Li et al. [34] reported similar results. They fabricated electrospun carbon electrodes for LIBs, and the best cycling performance with high capacity was observed at a carbonization temperature of 800 °C. However, a capacity decrease was observed with increasing temperature because of the more ordered structure at higher temperatures [34]. Tao et al. [35] observed the highest capacity for electrospun PVA-based CNFs at a carbonization temperature of 800 °C. At higher temperatures, the capacities decreased owing to the limited position of intercalations and the lowering of interlayer spacings [19,36,37].

The cycling performance for Na-ion cells containing CNFs carbonized at 700 °C, 800 °C, and 900 °C is shown in Figure 3H. The reversible capacities after 200 cycles for CNFs carbonized at 700 °C, 800 °C, and 900 °C were 87, 97, and 59 mAh/g, respectively. The good cyclability and high capacity could be attributed to the continuous structure of CNFs for fast electron transfer and large interlayer spacing between graphene layers, which is especially important for the storage properties of Na ions. Similarly, Xu et al. [38] reported a reversible capacity of approximately 100 mAh/g for electrospun CNF electrodes.

Developing a promising electrode material for Na-ion batteries is challenging because of its large ionic radius (ionic radius of Li^+^ = 76 nm and ionic radius of Na^+^ = 102 nm) [39]. Graphene, a two-dimensional (2D) carbonaceous material derived from graphite, is a promising candidate as an electrode in SIBs and demonstrates high electrical conductivity (~2000 S ${\mathrm{cm}}^{-1}$), good thermal conductivity (4840–5300 W$m^{-1}K^{-1}$), excellent cycle life, and flexibility due to many defects emerging from the residual oxygen-containing groups and its large surface area (~1500 $m^{2}g^{-1}$). In addition, the larger interlayer distance in graphene (0.37 nm) provides more active sites for the ion carriers compared to graphite (0.34 nm) [40].

### 3.3. Morphology, Structure, and Electrochemical Evaluation for MoS_2_-Decorated Graphene-Containing Porous Carbon Nanofiber

**Figure 4:**
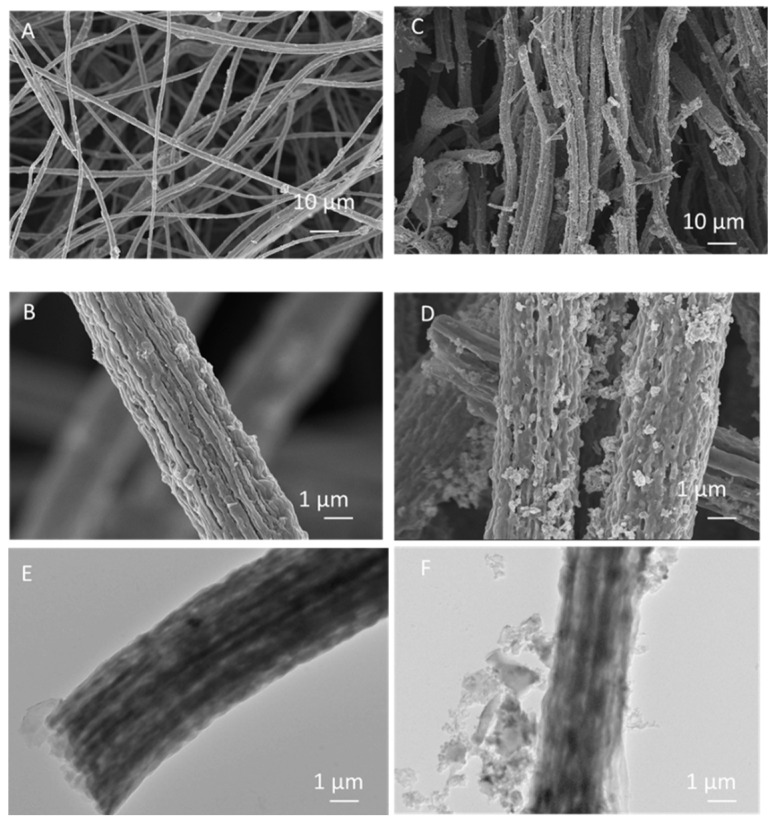
SEM images (**A**,**B**), of graphene@ porous carbon nanofiber and (**C**,**D**) MoS_2_-decorated graphene@ porous carbon nanofiber and TEM images of (**E**) graphene@ porous carbon nanofiber and (**F**) MoS_2_-decorated graphene@porous carbon nanofiber.

Increased specific surface areas can improve the electrochemical properties of electrodes and can be achieved by either decreasing the fiber diameter or creating pores in the fiber structure [18]. After determining the optimum carbonization temperature, graphene-containing porous CNFs were fabricated to improve the electrochemical properties of the CNF-based electrodes. Highly porous nanostructured carbons were prepared via centrifugal spinning of graphene@PAN/PS fibers followed by heat treatment. PS was used as a pore genarator. As seen from the SEM images (Figure 4A,B), CNFs were successfully obtained and surface roughness increased with graphene addition. MoS_2_ has high theoretical capacity and a graphene-like layered structure with large interlayer spacing of 0.62 nm. However, poor electronic conductivity and large volume change result in poor cycling performance [41]. MoS_2_ nanolayers were decorated on graphene@ porous carbon nanofibers via hydrothermal synthesis (Figure S1). Uniform MoS_2_ nanolayers anchored on graphene@ porous carbon nanofibers were seen from SEM in Figure 4C,D and Figure S2. Tubular structure with large amount of pores are also seen in TEM images of graphene@PCNFs (Figure 4E), while TEM images of MoS_2_-decorated G@PCNFs are proven MoS_2_-decorated highly porous nanostuctured carbons as seen in Figure 4F. A large specific surface area not only improves active sites for ion intercalation but also provides large area for the growth of high capacity active material. The growth of MoS_2_ on highly porous CNFs prevents agregation and improves cell kinetics via providing large surface area for electrochemical reactions.

The energy-dispersive X-ray spectroscopy (EDAX) analysis was carried out to determine the elements in the material. EDAX mapping of MoS_2_-decorated graphene@ porous carbon nanofibers is shown in Figure S3. We could observe the presence of molybdenum (Mo) and sulfur (S) atoms. Additionally, the presence of carbon (C) atoms for the MoS_2_-decorated graphene@ porous carbon nanofibers is clearly illustrated in Figure S3. Mapping images demonstrated that Mo atoms were uniformly dispersed on graphene@ porous carbon nanofibers. The highly uniform distribution of MoS_2_ at the nanometer level provided more active sites, which contributed to the electrochemical performance.

**Figure 5:**
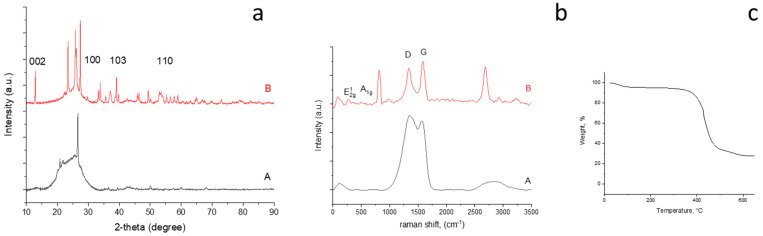
XRD pattern (**a**) and Raman spectra, D and G bands of carbon (**b**) of graphene@ porous carbon nanofiber (**A**), MoS_2_-decorated graphene@ porous carbon nanofiber (**B**), and TGA curve of MoS_2_-decorated on graphene@PCNFs (**c**).

XRD pattern and Raman spectra are shown in Figure 5. The XRD pattern of G@PCNFs consists of two peaks at approximately 26° and 43°, similar to that of the CNFs. The (002) peak became sharper and the crystalline size (L_c_) (23.85 nm) increased with the addition of graphene, which proves the presence of graphene. In the XRD pattern of MoS_2_-decorated G-PCNFs, the major peaks at 13.8°, 32.2°, 39.5°, and 58° are respectively indexed to the (002), (100), (103), and (110) planes of MoS_2_ [42], while minor peaks are ascribed to the crystalline planes of the hexagonal MoS_2_ phase (JCPDScardno.37-1492). The minor peaks at 33°, 43°, 50°, 55°, and 60° are ascribed to (101), (006), (105), (101), and (008), respectively [43,44,45].

The Raman spectra of G-PCNFs showed two peaks at 1347 cm^−1^ (D peak) and 1571 cm^−1^ (G peak) (Figure 5b). The amount of R (0.990) decreased slightly and L_a_ (4.44 nm) increased with graphene, which indicates a higher degree of order and thus higher ionic conductivity. The peaks at around 376.47 and 402.90 cm^−1^ were assigned to the E^1^_2g_ and A_1g_ modes of MoS_2_ [46].

The surface area and relative pore size of MoS_2_-decorated graphene@ porous carbon nanofiber were determined using Brunauer–Emmet–Teller (BET). The N_2_ adsorption–desorption isotherms and Barrett–Joyner–Halenda (BJH) plots are shown in Figure S2b,c. The average pore diameter was around 10 nm and a specific surface area of around 90 m^2^ g^−1^ was observed due to the highly porous carbon nanofibers.

TGA was used to determine MoS_2_ content in MoS_2_-decorated graphene@ porous carbon nanofibers. The TGA curve is shown in Figure 5c. There was a large amount of weight loss in the range of 400−500 °C, which was caused by the combustion of the amorphous carbon and a conversion of MoS_2_ to MoO_3_ in air. On the basis of the TGA results, the mass fraction of MoS_2_ was around 30%.

**Figure 6:**
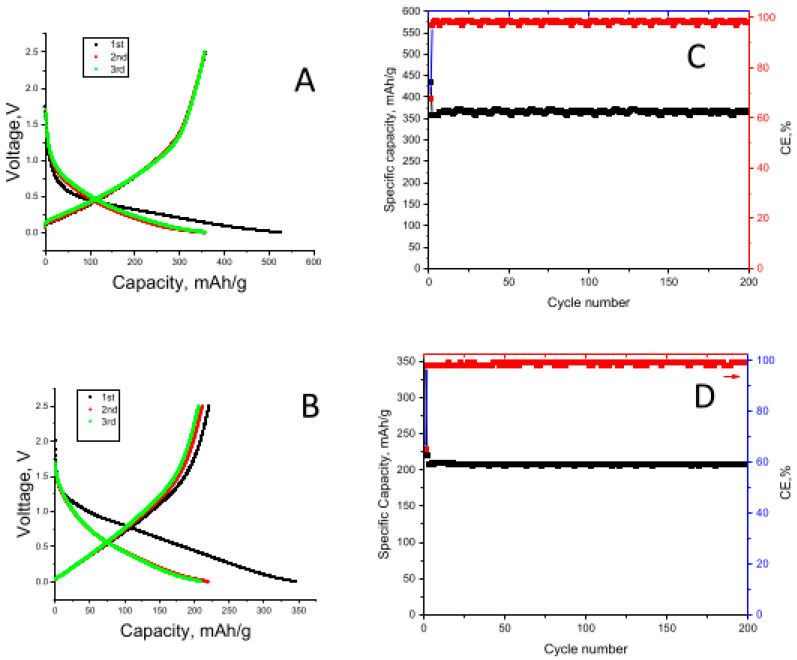
First-cycle discharge/charge curves and cycling performance of graphene@ porous carbon nanofiber electrodes in Li-ion (**A**,**C**) and Na-ion batteries (**B**,**D**).

The first-cycle discharge–charge curves and cycling performance of graphene@porous CNFs in Li-ion and Na-ion batteries are shown in Figure 6A–D, respectively. The first-cycle discharge–charge capacities were 523 mAh/g and 356 mAh/g in Li-ion cells and 345 and 221 mAh/g in Na-ion cells, respectively. The Coulombic efficiencies of the graphene@porous CNFs in Li- and Na-ion batteries were 68% and 64%, respectively, while the Coulombic efficiencies for the CNFs were 63% and 49%, respectively. Improved Coulombic efficiencies with the addition of graphene were reported by Dufficy et al. [11] alongside the low irreversible capacity values, which are explained by hindered side reactions between Li ions/electrolyte and residual oxygen and nitrogen compounds in the electrodes.

The reversible capacities were 365 mAh/g and 207 mAh/g for the Li-ion and Na-ion cells, respectively, in 200 cycles. The addition of graphene improved the electrochemical performance. Dufficy et al. [11] also reported improved capacities with the addition of graphene to electrospun CNFs, and the results were attributed to shortened Li-diffusion pathways and rapid charge transfer. Graphene acts as a conductive connector and further enhances the electron transport [11]. Wang et al. synthesized RGO using Hummer’s method [47] and used it as an electrode in SIBs. The prepared RGO electrode with an interlayer spacing of 0.371 nm for the (002) plane was favourable for accommodating large ${\mathrm{Na}}^{+}$ ions. After 250 cycles, it delivered a reversible capacity of 174.3 mAh/g and 93.3 mAh/g at 0.2 C and 1 C, respectively. This result was attributed to overlapping graphene nanosheets; thus, nanocavities, holes, and defects were formed, which is good for ${\mathrm{Na}}^{+}$-ion storage and decreased the diffusion length for ${\mathrm{Na}}^{+}$ ions [48].

**Figure 7:**
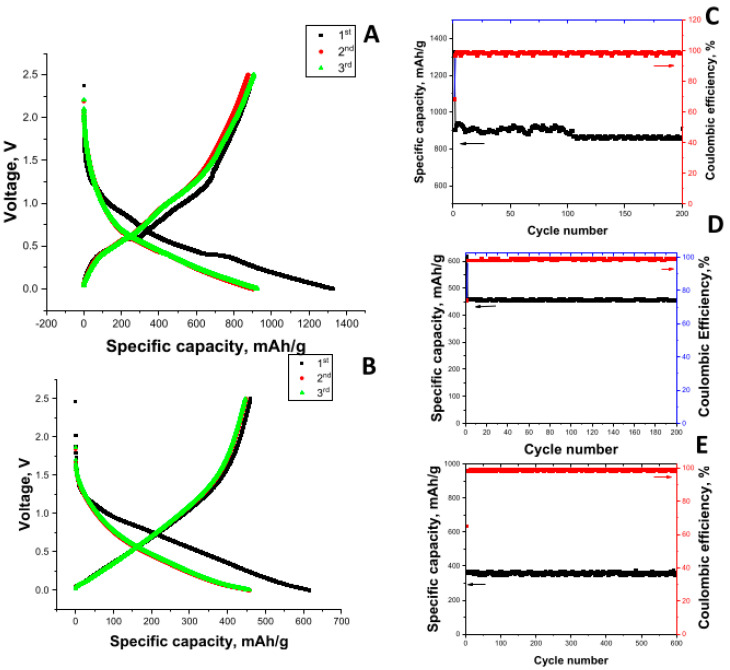
First-cycle discharge/charge curves and cycling performance of MoS_2_-decorated graphene@porous carbon nanofiber electrodes in Li-ion (**A**,**C**) and Na-ion batteries (**B**–**E**).

First-cycle discharge/charge curves and cycling performance of MoS_2_-decorated graphene@ porous carbon nanofiber electrodes in Li-ion (A,C) and Na-ion batteries (B,D) are seen in Figure 7. In Li-ion batteries, the first discharge capacity of 1326 mAh/g and charge capacity of 902 mAh/g were delivered, while reversible specific capacity was around 860 mAh/g in 200 cycles at 100 mA/g. In Na-ion batteries, the first discharge and charge capacities are 615 mAh/g and 458 mAh/g, respectively. Moreover, a reversible capacity around 455 mAh/g at 100 mA/g in 200 cycles was seen. Highly porous carbon nanofibers fabricated by using centrifugal spinning and heat treatment provided channels for continuous electron transfer on and along the fiber surface and improved wettability with electrolytes for fast ion diffusion. Furthermore, large surface areas provided more sites for the growth of MoS_2_ on PCNFs, which led to high specific capacity with excellent cycling stability. A cycling performance at 1000 mA/g is shown on Figure 7E. A reversible capacity over 380 mAh/g was seen in 600 cycles at 1 A/g. The highly porous structure with uniform nanostructured layer of MoS_2_ shortened the diffusion path of ions and improved cycling performance even at high C-rates. The inclusion of graphene not only improved the overall electronic conductivity but also contributed to storing more ions in the structure owing to the rougher surface observed from SEM and TEM images. Moreover, the high specific capacities and excellent cycling performance was attributed to the nanostructured MoS_2_ layer on highly porous graphene@CNFs with high conductivity. Graphene@ porous CNFs not only prevented aggregation of MoS_2_ but also provided more active sites for electrochemical reactions. Nanostructured active material coating on centrifugally spun highly porous carbon nanofibers with high conductivity provided short pathways for ion difusion and elecron transport as well as large contact areas between electrode and electrolyte, leading to excellent cycle performance even at high rates.

## 4. Conclusions

To support the development of cost-effective green energy storage in the face of climate change, this study explores the feasibility of nanostructured anodes in SIBs and LIBs. A novel electrode manufacturing and refinement process is presented in the form of a fast and reliable CNF preparation. Hydrothermal synthesis and heat treatment processes are presented, providing an efficient way to prepare high-performance battery electrodes. Additionally, the effect of the carbonization temperature during heat treatment on the structural properties and electrochemical performance of CNFs was investigated. XRD pattern and Raman spectra illustrated that increasing the carbonization temperature improved the ordered structure. CNFs carbonized at 800 °C, delivering the highest capacity compared to CNFs carbonized at 700 °C and 900 °C owing to high conductivity and large amount of defects. Furthermore, MoS_2_-decorated graphene-included porous CNF improved the electrochemical capacity up to 860 mAh/g in Li-ion cells and 455 mAh/g in Na-ion cells with excellent cycling performance. Combining centrifugal spinning and hydrothermal synthesis techniques is a promising way to fabricate high performance electrode materials.

## Data Availability

The data presented in this study are available on request from the corresponding author.

## Supplementary Materials

The following supporting information can be downloaded at: https://www.mdpi.com/article/10.3390/nano12142505/s1, Figure S1: Schematic of preparation of MoS_2_ decorated on graphene@PCNFs; Figure S2: SEM image (**a**), N2 adsorption–desorption isotherms (**b**) and BJH plots (**c**) of MoS_2_ decorated on graphene@PCNFs; Figure S3: EDX mapping of MoS_2_ decorated on graphene@PCNFs; Table S1: Interlayer spacing d(002), crystalline size Lc and in-plane graphitic crystalline size (La) of CNF-700, CNF-800, and CNF-900.
