# Supplementary material for: MoS2-Decorated Graphene@porous Carbon Nanofiber Anodes via Centrifugal Spinning
Source: Nanomaterials (Basel). 2022 Jul 21;12(14):2505. doi: 10.3390/nano12142505 (PMC9323995; doi:10.3390/nano12142505)
Supplement: Supplementary file 1 [file nanomaterials-12-02505-s001.zip › nanomaterials-1800305-supplementary.pdf]

nanomaterials

Supplementary Materials

MoS_2-Decorated Graphene@porous Carbon Nanofiber Anodes Via Centrifugal Spinning

Elham Abdolrazzaghian [1], Jiadeng Zhu [2], Juran Kim [3,*] and Meltem Yanilmaz [1,4,*]

1 Department of Nano Science and Nano Engineering, Istanbul Technical University, Istanbul 34469, Turkey; elham.a1992@gmail.com
2 Oak Ridge National Laboratory, Chemical Sciences Division, Oak Ridge, TN 37831, USA; jzhu14@ncsu.edu
3 Advanced Textile R&D Department, Korea Institute of Industrial Technology (KITECH), Ansan 15588, Korea
4 Department of Textile Engineering, Istanbul Technical University, Istanbul 34437, Turkey
* Correspondence: jkim0106@kitech.re.kr (J.K.); yanilmaz@itu.edu.tr (M.Y.)

Table S1. Interlayer spacing $d_{(002)}$, crystalline size L_c and in-plane graphitic crystalline size (L_a) of CNF-700, CNF-800 and CNF-900

Sample name	D(002), (nm)	Crystalline size Lc, (nm)	In-plane graphitic crystalline size (La), (nm)
CNF-700 °C	0.359	1.07	3.9
CNF-800 °C	0.353	1.11	4.4
CNF-900 °C	0.352	1.13	4.4

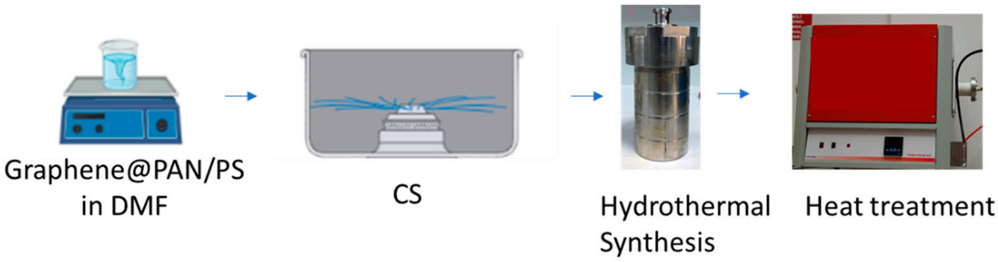

Figure S1. Schematic of preparation of MoS_2 decorated on graphene@PCNFs.

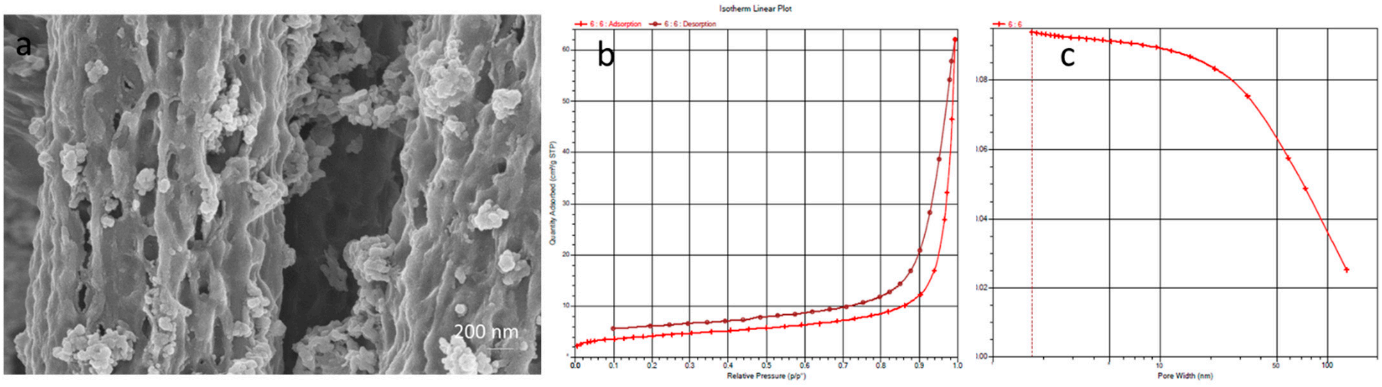

Figure S2. SEM image (**a**), N_2 adsorption -desorption isotherms (**b**) and BJH plots (**c**) of MoS_2 decorated on graphene@PCNFs.

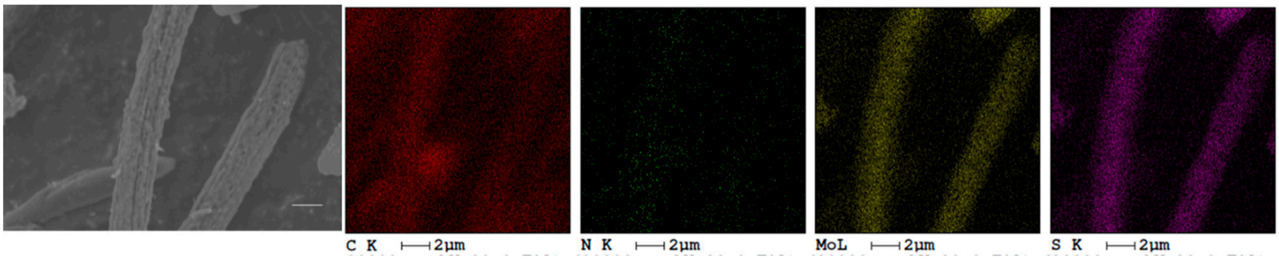

Figure S3. EDX mapping of MoS_2 decorated on graphene@PCNFs.
